# Pre-exposure to UV-B irradiation induces antioxidant enzymes and mitigates *Fusarium acutatum* stress in shallot (*Allium cepa* L.)

**DOI:** 10.1080/15592324.2026.2716981

**Published:** 2026-08-15

**Authors:** Febri Doni, Fanisa Miftah Riani, Muhammad Adil Awal, Dedat Prismantoro, Mia Miranti, Widhi Dyah Sawitri, Valentina Dwi Suci Handayani, Muhammad Idris, Sari Nurulita, Arif Wibowo, Ani Widiastuti

**Affiliations:** a Department of Biology, Faculty of Mathematics and Natural Sciences, Universitas Padjadjaran, Jatinangor, West Java, Indonesia; b Doctorate Program in Biotechnology, Graduate School, Universitas Padjadjaran, Bandung, West Java, Indonesia; c Department of Agronomy, Faculty of Agriculture, Universitas Gadjah Mada, Sleman, Yogyakarta, Indonesia; d Department of Biology, Faculty of Mathematics and Natural Sciences, Universitas Andalas, Padang, West Sumatra, Indonesia; e Department of Plant Protection, Faculty of Agriculture, IPB University, Bogor, West Java, Indonesia; f Department of Plant Protection, Faculty of Agriculture, Universitas Gadjah Mada, Sleman, Yogyakarta, Indonesia

**Keywords:** Antioxidant enzymes, defense priming, metabolomic profiling, phenolic compounds, sustainable disease management

## Abstract

Shallot (*Allium cepa* var. *aggregatum*) productivity is often limited by fungal diseases, particularly twisted disease caused by *Fusarium acutatum*. This study evaluated the effects of UV-B pre-exposure (150 min) on enhancing plant resistance under greenhouse conditions using four treatments: control, UV-B, *F. acutatum*, and UV-B + *F. acutatum*. UV-B pre-treatment significantly reduced disease incidence, severity, and the area under the disease progress curve (AUDPC) compared with infected plants without UV-B exposure. Although infection suppressed growth, UV-B pre-exposure alleviated these effects by maintaining higher plant height, root length, and biomass. UV-B also increased the photosynthetic pigment content, phenolic accumulation, and the activities of antioxidant enzymes, including catalase and peroxidase. LC–MS/MS metabolomic analysis revealed an increased abundance of metabolites associated with stress signaling, antioxidant responses, and defense-related metabolic pathways. The combined UV-B and pathogen treatment showed coordinated metabolic adjustments, indicating that UV-B priming enhances shallot tolerance to *F. acutatum* while sustaining growth, offering a sustainable disease management strategy.

## 1. Introduction

Shallot (*Allium cepa* var. *aggregatum*) is an economically important horticultural crop, with a global production of approximately 5.5 million metric tons in 2024, and it is recognized for its nutritional value and diverse bioactive compounds.^1^ Despite its high production potential, shallot yield and productivity are increasingly threatened by multiple agronomic and environmental challenges, including irregular rainfall patterns, drought stress, poor soil fertility, rising air temperatures, and the intensification of fungal and bacterial infections. Among these constraints, diseases caused by phytopathogenic infections are recognized as the primary factor responsible for significant yield losses in shallot cultivation.^2^ ^,^ ^3^ The increasing frequency and severity of these disease outbreaks further exacerbate production instability, particularly in intensive cultivation systems where disease pressure remains high.

One of the most destructive diseases affecting shallot production is twisted disease, which has been widely reported as a major cause of yield reduction. This disease is characterized by twisted and drooping leaves, which often progress to leaf and bulb rot and may ultimately result in plant death.^4^ ^,^ ^5^ The disease has been primarily attributed to infection by *Fusarium acutatum*, a soilborne phytopathogen capable of persisting in the field and causing recurrent outbreaks.^6^ The persistence of this pathogen in soil environments complicates disease management and limits the effectiveness of conventional control measures.

Plant disease prevention and management in horticultural cultivation have traditionally relied on the application of chemical fungicides.^3^ Although these control measures can reduce disease incidence in the short term, their prolonged and intensive use has been associated with adverse environmental impacts, including soil health degradation, environmental contamination, and the accelerated development of resistance in phytopathogenic populations.^7^ These limitations underscore the urgent need for alternative, sustainable disease management strategies. Consequently, the development of non-chemical approaches that enhance plant antioxidant defense responses and defense-associated physiological processes has become a priority in sustainable horticultural production.

Previous studies have demonstrated that ultraviolet-B (UV-B) irradiation represents a promising approach for suppressing the progression of phytopathogenic infections. As an environmentally friendly strategy, UV-B treatment functions as a pre-inductive stimulus that activates plant antioxidant defense mechanisms and defense-associated physiological responses prior to pathogen invasion. This defense priming is mediated, in part, through the activation of the *UV RESISTANCE LOCUS 8* (UVR8) signaling pathway, which plays a central role in UV-B perception by plants.^3^ ^,^ ^8^ Activation of the UVR8 pathway modulates defense-related gene expression and promotes the accumulation of protective secondary metabolites, thereby enhancing the plant’s capacity to resist subsequent pathogen infection.^8^ ^,^ ^9^

In this context, the present study examines a defined UV-B exposure regime, using a 150-minute irradiation period, as a pre-inductive approach to enhance resistance in shallots against *F. acutatum*. Plant growth, physiological characteristics, and biochemical defense markers were assessed to provide insight into the functional relevance of UV-B-mediated defense activation under controlled experimental conditions. Accordingly, this study aimed to determine the effectiveness of the 150-minute UV-B treatment in inducing resistance to *F. acutatum* and to characterize its associated growth, physiological, and biochemical responses in shallots.

## 2. Materials and methods

### 2.1. Fungal strains and isolate preparation

*F. acutatum* isolate SkmBP used in this study was obtained from the pathogenic fungal culture collection maintained by the Department of Plant Protection, Faculty of Agriculture, Universitas Gadjah Mada, Indonesia. The isolate was grown on potato dextrose agar (PDA) and incubated at room temperature for 7–10 d until the medium surface was fully covered by fungal growth, and visible mycelial development was observed.

After 7–10 d of incubation, when visible mycelial growth had developed, conidia of *F. acutatum* SkmBP were harvested. The conidia were collected by gently scraping the surface of the culture medium, followed by the addition of 10 mL of sterile distilled water.^10^ The conidial suspension was subsequently adjusted to a final density of 1 × 10^6^ spores mL^−1^.^3^ The number of *F. acutatum* SkmBP conidia was calculated according to the method described by Anhar et al.^11^ using the following formula:

Conidia Density:n×5×d/Vh

where

*n* = number of conidia;

d = dilution factor; and

Vh = hemocytometer volume.

### 2.2. Preparation of shallot plants

Shallots (*A. cepa* var., *aggregatum*) of the Bima Brebes cultivar were grown in 20 × 20  cm polybags containing a sterilized growth medium consisting of soil, manure, and litter. Only healthy and uniformly sized bulbs were selected (3–4 cm in diameter). Prior to planting, the upper one-third of each bulb was removed using a sterile knife, and the cut surfaces were treated with fungicide. Bulbs were planted upright with approximately three-quarters of the neck buried in the medium and watered twice daily, once in the morning and at noon. The cultivation was conducted under greenhouse conditions at the Plant Experimentation Station, Department of Biology, Universitas Padjadjaran. The plants at 40 d after planting were subsequently used for the in vivo experiment.

### 2.3. In vivo experiment

Shallot plants (cv. Bima Brebes) at 40 d after planting were used for the in vivo experiment. The study was arranged in a completely randomized design (CRD) with four treatments and ten replicates: (a) control, (b) UV-B irradiation, (c) UV-B irradiation combined with *F. acutatum* SkmBP inoculation (UV-B + F), and (d) *F. acutatum* SkmBP inoculation alone (F).

UV-B irradiation was applied using a UV-B lamp (Panasonic SPWFD24UB1PB) at an intensity of 25 µW cm^−2^ and a wavelength of 300 nm, delivering a total dose of 2.25 kJ m^−2^ s^−1^ for 150 min, following the procedures described by Widiastuti et al.^9^ For the UV-B + F and F treatments, a conidial suspension of *F. acutatum* SkmBP was applied to the rhizosphere at a volume of 10 mL per polybag, as described by Doni et al.^3^ and Awal et al.^2^ After treatment application, plants were maintained under greenhouse conditions for 18 d to allow disease development and plant responses to be evaluated.

During the experimental period, the environmental conditions were maintained at an average temperature of 30 ± 4 °C, light intensity of 320 ± 3 μmol m^−2^ s^−1^, relative humidity of 80 ± 3%, and a natural photoperiod of 11 h 11 min ± 9 s. Plant growth and disease symptoms were monitored periodically throughout the experimental period.

### 2.4. Disease incidence, disease severity index, and area under the disease progress curve

Disease development was evaluated using disease incidence (DI), the disease severity index (DSI), and the Area Under the Disease Progress Curve (AUDPC). Disease assessments were conducted every 3 d until 18 d after treatment following inoculation with *F. acutatum* isolate SkmBP, considering that twisted disease symptoms generally appear within 5–10 d after planting.^3^ The observed symptoms included root rot, bulb discoloration and softening, the presence of white fungal mycelia on bulbs, leaf twisting or yellowing, and abnormal basal plate elongation.^9^

Disease incidence (DI) was defined as the proportion of plants exhibiting disease symptoms and was calculated based on the number of symptomatic plants per polybag according to Wulan et al.^12^ using the following formula:

$$\mathrm{Disease incidence}\;\left(\mathrm{DI}\right)=\frac{\mathrm{number}\;\mathrm{of}\;\mathrm{infected}\;\mathrm{plants}}{\mathrm{total}\;n\mathrm{umber}\;of\;p\mathrm{lants}}\;\times 100\%$$

Disease severity was assessed following the methods of Wulan et al.^12^ and Doni et al.^3^ by assigning severity scores to each plant based on observed symptoms. The Disease Severity Index (DSI) was calculated to quantify disease intensity in each treatment using the following formula:

$$\mathrm{DSI}=\;\frac{\sum \left(n\times v\right)}{N\times Z}\;\times 100\%$$

Where,

*n* = number of plants showing a particular score;

*v* = disease severity score (0 = no symptoms; 1 = leaves wilted and yellowing; 2 = leaves yellowing and beginning to dry; 3 = leaves twisted; 4 = plants collapsed; and 5 = plants dead);

*N* = highest disease severity score, and Z = total number of plants observed.

The Area Under the Disease Progress Curve (AUDPC) was subsequently calculated based on disease severity data to describe disease progression over time and to estimate plant resistance under different treatments, following Wulan et al.^12^ and Campbell and Madden.^13^ The AUDPC value was determined using the following formula:

$$\mathrm{AUDPC}=\sum_{i=1}^{n-1}\;\left(\frac{Y_{i}+\;Y_{i+1}}{2}\right)\;(t_{i+1}-\;t_{i})$$

Notes: *N* = total number of observations; Y_i+1_ = disease severity score at the (i + 1)th observation; Y_i_ = disease severity score at the (i)th observation; t_i+1_ = time at the (i + 1)th observation; and t_i_ = time at the (i)th observation.

### 2.5. Plant morphological characteristics observation

Morphological observations were conducted based on the methods described by Altintas and Bal,^14^ with modifications. Observations were performed after completion of the in vivo experiment. The observed parameters included the number of leaves, plant height (cm), leaf length (cm), root length (cm), fresh weight (g), and dry weight (g). These measurements were used to evaluate plant growth responses under different treatment conditions.

### 2.6. Plant physiological characteristics observation

The physiological responses of shallot plants were assessed after the in vivo assay, including total phenolic content, antioxidant enzyme activity (catalase and peroxidase), and chlorophyll content.

#### 2.6.1. Total phenolic content

Total phenolic content was determined following Prismantoro et al.^15^ with slight modifications. Fresh leaf tissue (0.1 g) was homogenized in 10 mL of 70% acetone and centrifuged. A 1 mL aliquot of the supernatant was mixed with 1 mL of 1 N Folin–Ciocalteu reagent and 2 mL of 5% sodium carbonate, then diluted to 10 mL with distilled water. The mixture was heated until a blue color developed, and the absorbance was measured at 650 nm using a UV–Vis spectrophotometer. Gallic acid was used to generate the standard curve.

#### 2.6.2. Antioxidant enzyme analysis

Antioxidant enzymes, including catalase (CAT) and peroxidase (POD), were analyzed following Prismantoro et al.^15^ with modifications. The leaf samples were freeze-dried, ground into a fine powder, and stored at −80 °C until analysis. One gram of the powder was extracted with 10 mL of cold 50  mM potassium phosphate buffer (pH 6.8) and centrifuged at 12,000 × g for 20  min at 4 °C. The resulting supernatant was used as the enzyme extract. Catalase (CAT) activity was measured by mixing 50 µL of enzyme extract with 500 µL of 10 mM H₂O₂ and 2,450 µL of 50 mM potassium phosphate buffer, and the decrease in absorbance was recorded at 240 nm for 60 s. Peroxidase (POD) activity was determined by mixing 200 µL of enzyme extract with 1,000 µL of 100 mM potassium phosphate buffer, 500 µL of 20 mM guaiacol, and 300 µL of 10 mM H₂O₂, and the increase in absorbance was recorded at 470 nm at 5 s intervals for 90 s.

#### 2.6.3. Chlorophyll content

Chlorophyll content, including chlorophyll a, chlorophyll b, total chlorophyll, and carotenoids, was determined following Prismantoro et al.^15^ with slight modifications. Fresh leaf tissue (0.1 g) was immersed in 10 mL of 95% ethanol and incubated in the dark at 25 °C for 24 h until complete pigment extraction. The extract was then centrifuged, and the absorbance of the supernatant was measured at 663 nm, 645 nm, and 470 nm using a UV–Vis spectrophotometer. Chlorophyll and carotenoid contents were calculated using the following equations:

$$\mathrm{Chl}\;a={12.7A}_{663}–{2.69A}_{645}$$

$$\mathrm{Chl}\;b={22.9A}_{645}–{4.68A}_{663}$$

$$\mathrm{Total Chlorophyll}=\mathrm{Chl}\;a+\mathrm{Chl}\;b={20.2A}_{645}+{8.02A}_{663}$$

$$\mathrm{Caretonoids}=(1000\times A_{470}–1.82\times \mathrm{Chl}\;a–85.02\times \mathrm{Chl}\;b)/198$$

### 2.7. Metabolomic analysis of leaf samples using LC–MS/MS

The liquid chromatography–tandem mass spectrometry (LC–MS/MS) analytical procedures were conducted based on a modified method from Awal et al.^2^ Leaf samples were collected at 0 h (control), 6 h, 12 h, and 24 h after the final treatment. The samples were immediately frozen in liquid nitrogen and stored at −80 °C until extraction. Metabolites were extracted from 100 mg of frozen leaf tissue ground in liquid nitrogen and mixed with 1 mL of pre-cooled methanol:water (80:20, v/v) containing internal standards (ribitol, 60 µg mL^−1^ for GC–MS and genistein, 10 µg mL^−1^ for LC–MS). The extracts were sonicated for 20 min, centrifuged at 14,000 rpm for 10 min, and the supernatant was collected.

Data were processed using MetaboScape (LC–MS), followed by normalization to internal standards and total ion current (TIC). Metabolites were identified by comparison with the NIST library (GC–MS) and METLIN or KEGG databases (LC–MS). Fold-change (FC) analysis was performed using the relative peak area (%) of each putatively identified metabolite. FC values were calculated as the ratio of the relative peak area (%) of the treatment group to that of the corresponding reference group according to the following equation:

$$\mathrm{FC}=\;\frac{\mathrm{Area}\;(\%)\;\mathrm{of}\;\mathrm{Treatment}}{\mathrm{Area}\;(\%)\;\mathrm{of}\;\mathrm{Reference}}\;$$

where the reference group was either the control or the *F. acutatum*-treated plants, depending on the comparison (UV-B/Control, *F. acutatum*/Control, UV-B+*F. acutatum*/*F. acutatum*, or UV-B+*F. acutatum*/Control). Metabolites that were not detected (ND) in one or both groups were excluded from FC calculations.

### 2.8. Data analysis

Data analysis was performed according to the respective experimental methods. Qualitative data are presented as images, graphs, and tables, while quantitative data are presented in tables and graphical forms. The quantitative data were analyzed using analysis of variance (ANOVA) at a 95% confidence level using IBM SPSS Statistics 25. When significant differences were detected, Duncan’s Multiple Range Test (DMRT) at the 5% significance level was applied. In addition, Student’s t-test was applied for specific pairwise comparisons. To support the validity of the findings, the results were interpreted through comparison with relevant previous studies.

## 3. Results

### 3.1. Effect of UV-B pre-exposure on *F. acutatum*-induced twisted disease in shallot

**Figure 1.**
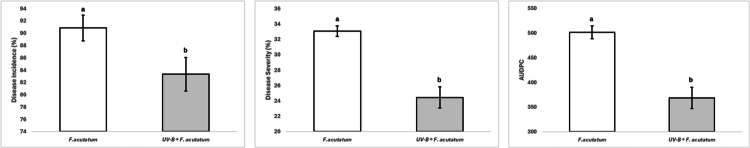
Effect of UV-B irradiation (150 min) on twisted disease development in shallot plants. Values are presented as mean ± standard error (SE) of four replicates. Data were collected 18 d after treatment. Different letters within the same column indicate significant differences between treatments (*p* < 0.05; Student's *t*-test).

The progression of twisted disease infection was evaluated by measuring disease incidence, disease severity, and the AUDPC (Figure 1). The typical symptoms of twisted disease observed in the shallot plants are indicative of *F. acutatum* infection, characterized by chlorosis and leaves failing to stand upright, instead exhibiting a rigid, horizontal twisting.

UV-B irradiation applied for 150 min significantly reduced all measured disease parameters compared to the non-irradiated control. The disease incidence in UV-B-treated plants decreased to 83.33%, compared with 90.84% in control plants. Similarly, disease severity was significantly lower in UV-B-treated plants (24.44%) than in the control plants (33.06%).

Furthermore, the AUDPC values, which represent disease progression over time, were markedly reduced following UV-B treatment. Plants exposed to UV-B irradiation exhibited an AUDPC value of 368.75, significantly lower than the control value of 501.25. These results demonstrate that UV-B pre-exposure effectively suppresses the development and progression of twisted disease in shallot plants. The consistent reduction in disease incidence, severity, and AUDPC indicates that UV-B treatment enhances plant resistance against *F. acutatum*, thereby limiting disease establishment and spread.

### 3.2. Effect of UV-B pre-exposure on morphological traits of shallot under *F. acutatum* infection

**Figure 2.**
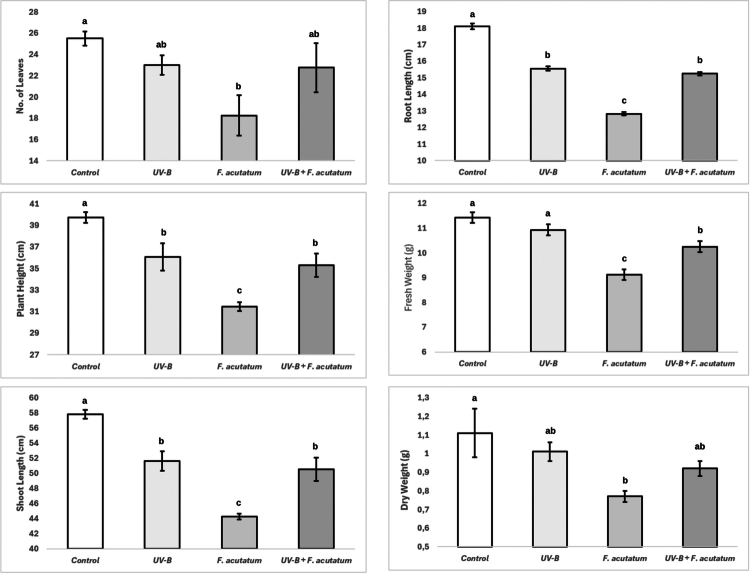
Morphological performance of shallot plants subjected to UV-B irradiation and *F. acutatum* inoculation. The values are presented as mean ± standard error (SE) of four replicates in the experiment. Data were recorded at 18 d after treatment. The same letter notation indicates an effect that is not significantly different according to the DMRT test (*P* < 0.05).

**Figure 3.**
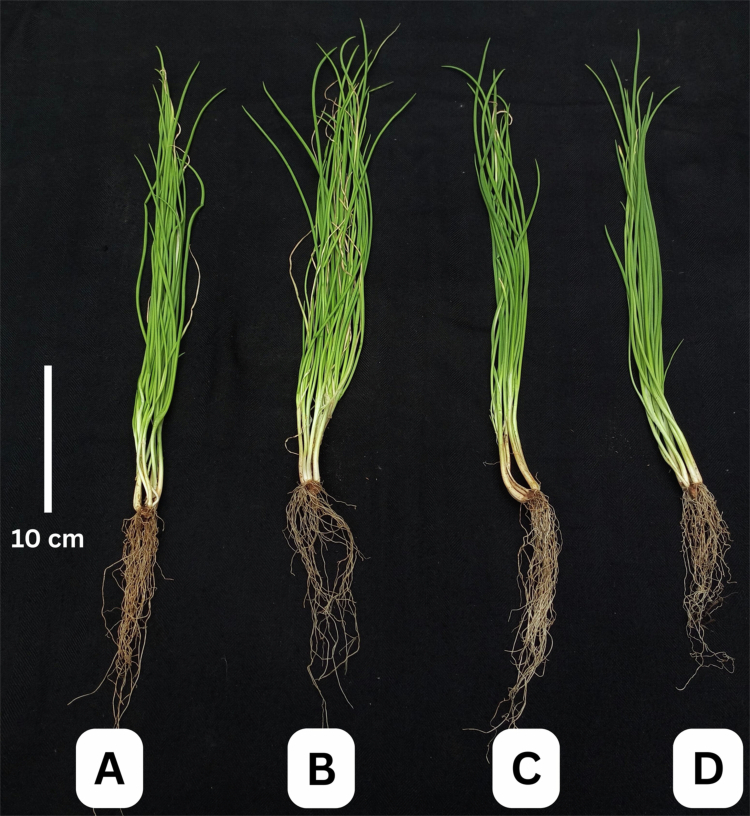
Morphological responses of shallot (*A. cepa* var. *aggregatum*) plants under different treatments: (A) control, (B) UV-B pre-exposure, (C) *F. acutatum* inoculation, and (D) UV-B pre-exposure followed by *F. acutatum* inoculation. Data were recorded at 18 d after treatment.

The effects of UV-B pre-exposure and *F. acutatum* infection on shallot morphological traits are presented in Figure 2. The visual differences in plant growth and root development among the treatments are shown in Figure 3. Significant differences among the treatments were observed across most growth parameters. In general, *F. acutatum* infection markedly reduced plant growth. Plants inoculated with *F. acutatum* alone (F) exhibited the lowest values for all measured parameters, including the number of leaves (18.25), plant height (31.46 cm), shoot length (44.28 cm), root length (12.83 cm), fresh weight (9.12 g), and dry weight (0.77 g). This finding indicates that pathogen infection severely inhibited both vegetative growth and biomass accumulation.

In contrast, control plants (C) showed the highest values across all parameters, reflecting optimal growth under non-stress conditions. UV-B treatment alone (UV-B) did not negatively affect plant growth and maintained comparable values to the control for several parameters, particularly fresh weight and dry weight, which were not significantly different. Notably, UV-B pre-exposure mitigated the adverse effects of *F. acutatum* infection. Plants subjected to the combined treatment (UV-B+F) showed significantly higher values for plant height, shoot length, root length, and biomass compared to plants inoculated without UV-B (F). For instance, plant height increased from 31.46 cm (F) to 35.30 cm (UV-B+F), while root length improved from 12.83 to 15.25 cm. These findings suggest that UV-B pre-exposure plays a protective role in maintaining plant growth under pathogen stress. The ability of UV-B treatment to alleviate growth inhibition caused by *F. acutatum* indicates its potential to enhance plant tolerance by sustaining morphological development and biomass production.

### 3.3. Induction of antioxidant responses in shallot by UV-B pre-exposure under *F. acutatum* infection

#### 3.3.1. Effect of UV-B pre-exposure on photosynthetic pigments

**Figure 4.**
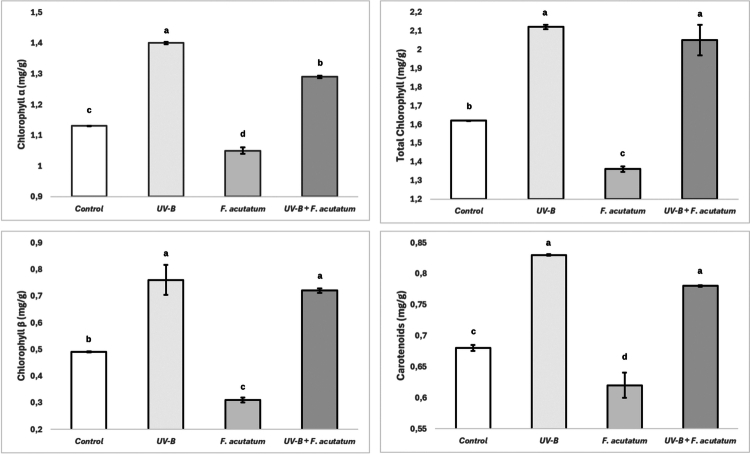
Effect of UV-B pre-exposure and *F. acutatum* infection on the photosynthetic pigment contents in shallot plants. The values are presented as mean ± standard error (SE) of three replicates in the experiment. Data were recorded at 18 d after treatment. The same letter notation indicates an effect that is not significantly different according to the DMRT test (*P* < 0.05).

UV-B pre-exposure significantly influences photosynthetic pigment accumulation in shallot plants (Figure 4). The plants exposed to UV-B alone exhibited the highest levels of chlorophyll *a* (1.40 mg/g), chlorophyll *b* (0.76 mg/g), total chlorophyll (2.12 mg/g), and carotenoids (0.83 mg/g), all of which were significantly greater than those observed in the control treatment (*P* < 0.05).

In contrast, *F. acutatum* infection markedly reduced the pigment content, with the lowest values recorded for chlorophyll *a* (1.05 mg/g), chlorophyll *b* (0.31 mg/g), total chlorophyll (1.36 mg/g), and carotenoids (0.62 mg/g). Notably, plants subjected to combined UV-B pre-exposure and pathogen infection (UV-B+*F. acutatum*) showed a significant recovery in pigment levels compared to pathogen treatment alone.

The total chlorophyll (2.05 mg/g) and chlorophyll *b* (0.72 mg/g) contents in UV-B+*F. acutatum*-treated plants did not differ significantly from those in UV-B-treated plants (2.12 and 0.76 mg/g, respectively), indicating that UV-B pre-exposure effectively alleviated the adverse effects of *F. acutatum* infection on photosynthetic pigment accumulation.

#### 3.3.2. Effect of UV-B pre-exposure on total phenolic content

**Figure 5.**
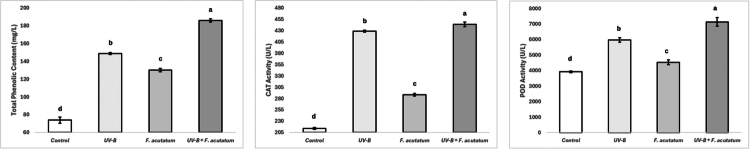
Effect of UV-B pre-exposure and *F. acutatum* infection on total phenolic content and antioxidant enzyme activities in shallot plants. The values are presented as mean ± standard error (SE) of three replicates in the experiment. Data were recorded at 18 d after treatment. The same letter notation indicates an effect that is not significantly different according to the DMRT test (*P* < 0.05).

The total phenolic content was significantly affected by both UV-B treatment and pathogen infection (Figure 5). UV-B pre-exposure alone resulted in a substantial increase in phenolic content (148.60 mg/L) compared to the control (73.80 mg/L). Interestingly, *F. acutatum* infection also induced phenolic accumulation (130.07 mg/L), although to a lesser extent than UV-B treatment. The highest phenolic content was observed in the combined UV-B + *F. acutatum* treatment (185.77 mg/L), which was significantly greater than all other treatments (*P* < 0.05). This synergistic increase suggests that UV-B pre-exposure primes antioxidant and defense-associated physiological responses, leading to enhanced production of phenolic compounds upon pathogen challenge.

#### 3.3.3. Effect of UV-B pre-exposure on antioxidant enzymes

The activities of antioxidant defense enzymes, CAT and POD, were significantly enhanced by UV-B pre-exposure and pathogen infection (Figure 5). UV-B treatment alone significantly increased the CAT (428.50 U/L) and POD (5,970.00 U/L) activities compared to the control (213.90 U/L and 3,913.33 U/L, respectively). Similarly, pathogen infection elevated enzyme activities, although to a lesser extent than UV-B treatment. The highest enzyme activities were recorded in the combined UV-B+*F. acutatum* treatment, with CAT reaching 443.40 U/L and POD 7,130.00 U/L. These values were significantly higher than those observed in all other treatments (*P* < 0.05). The pronounced increase in antioxidant enzyme activities under the combined treatment indicates a synergistic effect of UV-B pre-exposure and pathogen stress, enhancing the plant’s capacity to detoxify reactive oxygen species and improve stress tolerance.

### 3.4. LC–MS/MS-based profiling of non-volatile metabolites in shallot under UV-B pre-exposure and *F. acutatum* infection

**Figure 6.**
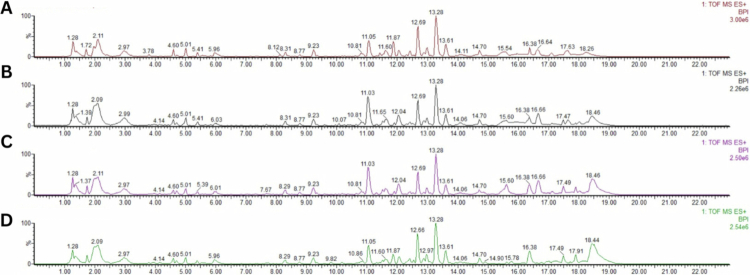
LC-MS/MS chromatogram of shallot plants under different treatments: (A) control, (B) UV-B pre-exposure, (C) *F. acutatum* inoculation, and (D) UV-B pre-exposure followed by *F. acutatum* inoculation.

Non-volatile metabolites in shallot leaves were profiled using LC–MS/MS to investigate biochemical responses associated with UV-B pre-exposure and *F. acutatum* infection. Representative chromatograms obtained from different treatments are shown in Figure 6, demonstrating qualitative differences in metabolite composition among the control, UV-B-treated, pathogen-inoculated, and combined treatment groups.

Qualitative differences in peak intensity and distribution were observed across treatments, indicating treatment-dependent changes in metabolite composition. Compared with the control, UV-B-treated plants showed a greater abundance of metabolites previously reported to be associated with stress signaling and antioxidant-related responses. In contrast, plants inoculated with *F. acutatum* exhibited a metabolite profile enriched in compounds that have been reported to possess antimicrobial or antifungal properties.

Relative peak area (%) and fold-change (FC) analyses of the annotated metabolites are presented in Supplementary Table S1. Several metabolites exhibited treatment-dependent differences in abundance. Compared with that of the control, the carbonic acid derivative detected at RT 1.28 increased approximately fourfold following UV-B treatment (FC = 4.06) and remained elevated in the combined UV-B+*F. acutatum* treatment (FC = 3.61). The purine nucleoside derivative detected at RT 2.97 and the polyamine derivative detected at RT 5.01 also showed higher abundance following UV-B treatment (FC = 1.23 for both compounds). In contrast, the amino-sugar conjugate detected at RT 12.66 decreased following UV-B treatment (FC = 0.73), whereas the quinazoline derivative detected at RT 11.60 increased under pathogen infection (FC = 1.24 relative to the control) and showed a further increase in the combined treatment (FC = 1.32 relative to the pathogen treatment). Several metabolites were detected only under specific treatments, including a peptide-like compound detected at RT 11.87 that was observed exclusively in the UV-B+*F. acutatum* treatment.

The functional classification of the identified metabolites (Table 1) further highlighted treatment-specific differences in metabolite composition. UV-B pre-exposure resulted in the highest number of metabolites associated with stress signaling and defense-associated responses (four compounds), whereas *F. acutatum* infection alone showed the greatest number of metabolites reported to possess antimicrobial or antifungal activity (seven compounds). These differences indicate that UV-B pre-exposure and pathogen infection were associated with distinct metabolic responses.

**Table 1.** Functional classification of metabolites identified in shallot leaves under UV-B irradiation and *F. acutatum* infection.

Functions	Control	UV-B	*F. acutatum*	UV-B+*F. acutatum*
Induction of plant defense (biotic and abiotic stress signaling)	2	4	2	3
Stress signaling and metabolic regulation (general)	2	2	2	2
Osmoprotection/redox modulation	1	–	–	1
Cell wall reinforcement/immune modulation	1	1	–	1
Direct antimicrobial/antifungal activity	6	4	7	6
Dual function (antimicrobial + defense/plant-related)	1	1	–	1
Plant growth-related/bioactive peptide	1	–	–	1
Unknown/not clearly defined function	4	6	3	4

Interestingly, the combined UV-B+*F. acutatum* treatment exhibited metabolites associated with both stress signaling and antimicrobial activity, reflecting the characteristics observed in the individual UV-B and pathogen treatments. A diverse range of nitrogen-containing metabolites, polyamine derivatives, peptide-like compounds, amino-sugar conjugates, and phenolic-related structures was identified across all treatments (Table 2). Several of these metabolite classes have previously been reported to participate in stress signaling, redox regulation, cell-wall reinforcement, and antimicrobial responses. In particular, polyamine derivatives and amino-sugar conjugates detected in the UV-B and combined treatments have been associated with antioxidant regulation and stress adaptation, whereas quinazoline, benzimidazole, and hydrazine derivatives detected predominantly in pathogen-inoculated plants have been reported to possess antimicrobial or antifungal properties. The occurrence of these metabolites, together with the enhanced CAT and POD activities and increased phenolic content observed in UV-B-treated plants, indicates that UV-B pre-exposure was associated with coordinated physiological and metabolic responses that may contribute to reduced disease development.

**Table 2.** List of non-volatile compounds successfully identified from liquid chromatography–mass spectrometry (LC–MS) analysis.

No	Retention Times	Compounds	Formulas	Area (%)	Molecular weight (g/mol)	Functions	References
**Control**							
1	1.28	carbonic acid;2-ethyl-2-(hydroxymethyl)propane-1,3-diol	C_9_H_20_O_12_	1.59	381.08	It contains a diol functional group, which can play a role in antimicrobial activity	Iwasaki et al. [16]
2	2.09	3-[2-[2-[(2-hydrazinyl-2-oxoethyl)carbamoyl]hydrazinyl]-2-oxoethyl]-1,1-dimethylurea	C₈H₁₇N₇O₄	15.67	276.15	Known to inhibit fungal growth and act as hydrazinyl antifungal	Wang et al. [17]
3	2.97	[(2 R, 3S, 4 R, 5 R)-5-(6-aminopurin-9-yl)-3, 4-dihydroxyoxolan-2-yl]methylurea	C₁₁H₁₅N₇O₄	4.63	310.13	A purine nucleoside derivative that can act as a signaling molecule involved in plant stress responses, metabolic regulation, and defense-related communication	Li et al. [18]
4	4.60	6, 10-dimethyldodecan-6-ol	C₁₄H₂₉O	1.40	340.26	N/A	N/A
5	5.01	1-[N'-[3-[4-[3-[[amino-(prop-2-enylcarbamoylamino)methylidene]amino]propylamino]butylamino]propyl]carbamimidoyl]-3-prop-2-enylurea	C₂₀H₄₀N₁₀O₂	1.16	453.34	A nitrogen-rich polyamine derivative potentially involved in stress signaling, polyamine-homeostasis modulation, and priming of defense responses.	Napieraj et al. [19]
6	5.39	hexadecyl(dipropyl)oxidanium	C₂₂H₄₇O	0.86	566.43	N/A	N/A
7	8.29	4-(4-pentylcyclohexyl)benzoic acid	C₁₈H₂₆O₂	1.16	275.20	A benzoic acid derivative associated with phenolic metabolism; may contribute to stress-induced signaling or membrane-related defense responses.	Windisch et al. [20]
8	11.60	7-ethyl-6-methoxy-2-(4-methyl-1,4-diazepan-1-yl)-*N*-(1-methylpiperidin-4-yl)quinazolin-4-amine	C₂₃H₃₆N₆O	1.79	413.31	Quinazoline structures are reported to suppress fungal growth	Liu et al. [21]
9	11.87	(2S)-*N*-[5-[[(2S)-2-amino-6-[3-[[(2S)-2-amino-5- diaminomethylideneamino)pentanoyl]amino]propylamino]hexanoyl]amino]pentyl]-2-[[2-(1H-indol-3-yl)acetyl]amino]butanediamide	C₃₄H₅₈N₁₂O₅	3.61	715.47	Belongs to peptide-based compounds with antifungal and plant growth-promoting properties	Song et al. [22]
10	12.66	*N*-[6-[3,5-diamino-2-[3-amino-6-(aminomethyl)-4,5-dihydroxyoxan-2-yl]oxy-6-hydroxycyclohexyl]oxyhexyl]-4-oxopentanamide	C₂₃H₄₅N₅O₈	6.13	520.34	A complex amino-sugar conjugate that may contribute to cell-wall reinforcement and immune signaling, as glycosylated amine derivatives are known to modulate defense via plant glycan recognition	Zhang et al. [23]
11	13.28	4-hydroxy-*N*-[5-[hydroxy-[1-hydroxy-4-[5-[hydroxy(1-hydroxyethyl)amino]pentylamino]-4-oxobutyl]amino]pentyl]-4-[hydroxy(methyl)amino]butanamide	C₂₁H₄₅N₅O₈	10.66	496.34	A polar amino-sugar-polyol derivative that may function as an osmoprotectant and redox modulator, and potentially act in sugar-amine immune signaling	Zhang et al. [23]
12	16.38	(2 R)-1-[[(1S, 2S)-2-[[(2 R)-2-hydroxy-9-phenylnonyl]-methylamino]cyclohexyl]-methylamino]-9-phenylnonan-2-ol	C₃₈H₆₂N₂O₂S	4.47	611.47	N/A	N/A
13	17.49	18-[2-[2-[2-[2-[2-[2-[[(5S)-5-carboxy-5-[[3-[3-(1H-imidazol-5-yl)propanoylamino]-3-methyl-2-oxobutyl]amino]pentyl]amino]-2-oxoethoxy]ethoxy]ethylamino]-2-oxoethoxy]ethoxy]ethylamino]-18-oxooctadecanoic acid	C₄₇H₈₃N₇O₁₃	1.70	954.61	Functions as a lipopeptide-like compound that may contribute to antifungal activity through interaction with and disruption of fungal cell membranes	Liu et al. [21]
14	17.91	tert-butyl 4-[11-[[(2S)-5-[(2-methylpropan-2-yl)oxy]-2-[4-[5-[(2-methylpropan-2-yl)oxycarbonylamino]pentyl]triazol-1-yl]-5-oxopentanoyl]amino]undecanoyl]piperazine-1-carboxylate	C₄₁H₇₃N₇O₈	1.43	792.56	Piperazine derivatives are known for antifungal activity	Kharb et al. [24]
15	18.44	ethyl 4-[[6-[bis(3-methylbutyl)carbamoyl]-1-[3-[methyl(pentyl)amino]propyl]benzimidazol-2-yl]amino]piperidine-1-carboxylate	C₃₅H₆₀N₆O₃	17.92	613.48	Contains benzimidazole-like potential antifungal mechanism	Marinescu et al. [25]
**UV-B**							
1	1.28	carbonic acid;2-ethyl-2-(hydroxymethyl)propane-1, 3-diol	C₉H₂₀O₁₂	6.46	381.08	It contains a diol functional group, which can play a role in antimicrobial activity	Iwasaki et al. [16]
2	2.09	3-[2-[2-[(2-hydrazinyl-2-oxoethyl)carbamoyl]hydrazinyl]-2-oxoethyl]-1, 1-dimethylurea	C₈H₁₇N₇O₄	17.28	276.15	Known to inhibit fungal growth and act as hydrazinyl antifungal	Chaki et al. [26]
3	2.99	[(2 R, 3S, 4 R, 5 R)-5-(6-aminopurin-9-yl)-3, 4-dihydroxyoxolan-2-yl]methylurea	C₁₁H₁₅N₇O₄	5.69	310.13	A purine nucleoside derivative that can act as a signaling molecule involved in plant stress responses, metabolic regulation, and defense-related communication	Li et al. [18]; Witte et al. [27]
4	4.60	6, 10-dimethyldodecan-6-ol	C₁₄H₂₉O	1.30	340.26	N/A	N/A
5	5.01	1-[N'-[3-[4-[3-[[amino-(prop-2-enylcarbamoylamino)methylidene]amino]propylamino]butylamino]propyl]carbamimidoyl]-3-prop-2-enylurea	C₂₀H₄₀N₁₀O₂	1.43	453.34	A nitrogen-rich polyamine derivative potentially involved in stress signaling, polyamine-homeostasis modulation, and priming of defense responses	Napieraj et al. [19]
6	5.39	hexadecyl(dipropyl)oxidanium	C₂₂H₄₇O	0.76	566.43	N/A	N/A
7	8.31	1-[[1-[3-(1H-imidazol-5-yl)propyl]triazol-4-yl]methyl]piperidine	C₁₄H₂₂N₆	1.05	275.20	Enhanced antifungal activity through the piperidine side chain	Jangir et al. [28]
8	12.04	*N*-[3-[4-[amino(3-aminopropyl)amino]butyl-sulfanylamino]propyl]-2-hydrazinyl-*N*-methylpropanamide	C₁₄H₃₅N₇O	2.92	318.30	Hydrazine derivatives are known to possess antimicrobial and antifungal properties, suggesting a possible contribution to the inhibition of Fusarium growth.	Wang et al. [17]
9	12.69	*N*-[6-[3, 5-diamino-2-[3-amino-6-(aminomethyl)-4, 5-dihydroxyoxan-2-yl]oxy-6-hydroxycyclohexyl]oxyhexyl]-4-oxopentanamide	C₂₃H₄₅N₅O₈	4.46	520.34	A complex amino-sugar conjugate that may contribute to cell-wall reinforcement and immune signaling, as glycosylated amine derivatives are known to modulate defense via plant glycan recognition	Zhang et al. [23]
10	13.28	(2 R, 4S, 5S, 7S)-5-amino-*N*-(3-amino-3-oxopropyl)-4-hydroxy-7-[[4-methoxy-3-(3-methoxypropoxy)phenyl]methyl]-2, 8-dimethylnonanamide	C₂₆H₄₅N₃O₆	9.68	496.34	N/A	N/A
11	15.6	*N*-[(2S)-1-[[(2S)-1-[[(2S)-1-amino-5-(diaminomethylideneamino)-1-oxopentan-2-yl]amino]-5-(diaminomethylideneamino)-1-oxopentan-2-yl]amino]-5-(diaminomethylideneamino)-1-oxopentan-2-yl]tetradecanamide	C₃₂H₆₅N₁₃O₄	7.07	696.54	Reported as guanidine–peptide derivative with antifungal and defense-inducing activity	Nawrot et al. [29]
12	16.38	[(2S)-1-hydroxy-3-pentadecanoyloxypropan-2-yl] (9Z, 12Z)-octadeca-9, 12-dienoate	C₃₆H₆₆O₅S	3.48	611.47	Fatty acid derivatives are also known to participate in plant defense signaling pathways and may contribute to induced resistance against pathogens	Chanthini et al. [30]
13	16.66	3-[[(1, 3-diethylimidazolidin-2-ylidene)amino]carbamoylamino]-4-methoxy-*N*-methyl-*N*-octadecylbenzamide	C₃₅H₆₂N₆O₃	4.31	615.50	Structural features associated with antifungal activity have been reported	Sulistyowaty et al. [31]
14	17.63	2-[9-(1, 4, 8, 11-tetrazacyclotetradec-2-yl)-1, 4, 7, 10-tetrazacyclododec-2-yl]-3, 6, 9, 15-tetrazabicyclo[9.3.1]pentadeca-1(14), 11(15), 12-triene	C₂₉H₅₈N₁₂	1.88	575.50	N/A	N/A
15	18.46	ethyl 4-[[6-[bis(3-methylbutyl)carbamoyl]-1-[3-[methyl(pentyl)amino]propyl]benzimidazol-2-yl]amino]piperidine-1-carboxylate	C₃₅H₆₀N₆O₃	6.62	613.48	Contains benzimidazole-like potential antifungal mechanism	Marinescu et al. [25]
***F. acutatum***							
1	1.28	carbonic acid;2-ethyl-2-(hydroxymethyl)propane-1,3-diol	C₉H₂₀O₁₂	6.51	381.08	It contains a diol functional group, which can play a role in antimicrobial activity	Iwasaki et al. [16]
2	2.11	3, 9-bis(azidomethyl)-1, 5, 6, 7, 12-pentazatricyclo[7.3.0.03, 7]dodeca-5, 11-diene	C₉H₁₃N₁₁	14.62	276.15	High-nitrogen structure linked to RNS-based defense signaling	Haring et al. [32]
3	2.97	[(2 R, 3S, 4 R, 5 R)-5-(6-aminopurin-9-yl)-3, 4-dihydroxyoxolan-2-yl]methylurea	C₁₁H₁₅N₇O₄	4.56	310.13	A purine nucleoside derivative that can act as a signaling molecule involved in plant stress responses, metabolic regulation, and defense-related communication	Li et al. [18]; Witte et al. [27]
4	4.60	6, 10-dimethyldodecan-6-ol	C₁₄H₂₉O	1.32	340.26	N/A	N/A
5	8.29	1-[[1-[3-(1H-imidazol-5-yl)propyl]triazol-4-yl]methyl]piperidine	C₁₄H₂₂N₆	1.32	275.20	Enhanced antifungal activity through the piperidine side chain	Jangir et al. [28]
6	11.60	7-ethyl-6-methoxy-2-(4-methyl-1, 4-diazepan-1-yl)-*N*-(1-methylpiperidin-4-yl)quinazolin-4-amine	C₂₃H₃₆N₆O	2.22	413.31	Quinazoline structures are reported to suppress fungal growth	Liu et al. [21]
7	12.04	*N*-[3-[4-[amino(3-aminopropyl)amino]butyl-sulfanylamino]propyl]-2-hydrazinyl-*N*-methylpropanamide	C₁₄H₃₅N₇O	3.38	318.30	Hydrazine derivatives are known to possess antimicrobial and antifungal properties, suggesting a possible contribution to the inhibition of Fusarium growth.	Wang et al. [17]
8	13.28	2-aminoethyl *N*-[6-amino-1-(2-carbamoyloxyethylcarbamoyloxy)hexan-2-yl]carbamate;butan-1-amine;propanamide	C₂₀H₄₅N₇O₇	10.26	496.34	A nitrogen-rich polyamine-carbamate-type derivative that may influence plant nitrogen‐metabolism signaling and modulate stress responses, including defense priming and enhanced tolerance.	Minocha et al. [33]
9	15.60	*N*-[1-[[(3 R, 4S, 5S)-3-methoxy-1-[2-[(1 R, 2 R)-1-methoxy-2-methyl-3-oxo-3-(2-piperazin-1-ylethylamino)propyl]pyrrolidin-1-yl]-5-methyl-1-oxoheptan-4-yl]-methylamino]-3-methyl-1-oxobutan-2-yl]-3-methyl-2-(methylamino)butanamide	C₃₆H₆₉N₇O₆	5.71	696.54	Contains piperazine, associated with antifungal and antimicrobial properties	Zhang et al. [34]
10	16.38	(2 R)-1-[[(1S, 2S)-2-[[(2 R)-2-hydroxy-9-phenylnonyl]-methylamino]cyclohexyl]-methylamino]-9-phenylnonan-2-ol	C₃₈H₆₂N₂O₂S	3.05	611.47	N/A	N/A
12	16.66	3-[[(1, 3-diethylimidazolidin-2-ylidene)amino]carbamoylamino]-4-methoxy-*N*-methyl-*N* octadecylbenzamide	C₃₅H₆₂N₆O₃	5.14	615.50	Structural features associated with antifungal activity have been reported	Sulistyowaty et al. [31]
13	18.46	ethyl 4-[[6-[bis(3-methylbutyl)carbamoyl]-1-[3-[methyl(pentyl)amino]propyl]benzimidazol-2-yl]amino]piperidine-1-carboxylate	C₃₅H₆₀N₆O₃	11.89	613.48	Contains benzimidazole-like potential antifungal mechanism	Marinescu et al. [25]
**UV-B+** ***F. acutatum***							
1	1.28	carbonic acid;2-ethyl-2-(hydroxymethyl)propane-1.3-diol	C₉H₂₀O₁₂	5.74	381.08	It contains a diol functional group, which can play a role in antimicrobial activity	Iwasaki et al. [16]
2	2.11	methyl 8-acetyloxy-7-nitrononanoate	C₁₂H₂₁NO₆	11.63	276.15	Nitro-fatty acid derivatives could play a role in abiotic and biotic stress signaling	Chaki et al. [26]
3	2.97	*N*'-[3-[(*Z*)-[amino(pyrazin-2-yl)methylidene]amino]-1*H*-1.2.4-triazol-5-yl]pyrazine-2-carboximidamide	C₁₂H₁₁N₁₁	4.65	310.13	May function as triazole antifungal and nitrogen-rich heterocycle with antifungal potential	Wang et al. [17]
4	4.60	6.10-dimethyldodecan-6-ol	C₁₄H₂₉O	1.64	340.26	N/A	N/A
5	5.01	1-[N'-[3-[4-[3-[[amino-(prop-2-enylcarbamoylamino)methylidene]amino]propylamino]butylamino]propyl]carbamimidoyl]-3-prop-2-enylurea	C₂₀H₄₀N₁₀O₂	1.70	453.34	A nitrogen-rich polyamine derivative potentially involved in stress signaling, polyamine-homeostasis modulation, and priming of defense responses	Napieraj et al. [19]; Sheng et al. [35]
6	5.39	hexadecyl(dipropyl)oxidanium	C₂₂H₄₇O	1.16	566.43	N/A	N/A
7	11.60	7-ethyl-6-methoxy-2-(4-methyl-1.4-diazepan-1-yl)-*N*-(1-methylpiperidin-4-yl)quinazolin-4-amine	C₂₃H₃₆N₆O	2.92	413.31	Quinazoline structures are reported to suppress fungal growth	Liu et al. [21]
8	11.87	(2S)-*N*-[5-[[(2S)-2-amino-6-[3-[[(2S)-2-amino-5-(diaminomethylideneamino)pentanoyl]amino]propylamino]hexanoyl]amino]pentyl]-2-[[2-(1H-indol-3-yl)acetyl]amino]butanediamide	C₃₄H₅₈N₁₂O₅	5.81	715.47	Belongs to peptide-based compounds with antifungal and plant growth-promoting properties	Song et al. [22]
9	12.04	*N*-[3-[4-[amino(3-aminopropyl)amino]butyl-sulfanylamino]propyl]-2-hydrazinyl-*N*-methylpropanamide	C₁₄H₃₅N₇O	0.98	318.30	Hydrazine derivatives are known to possess antimicrobial and antifungal properties, suggesting a possible contribution to the inhibition of *Fusarium* growth	Wang et al. [17]
10	12.69	*N*-[6-[3.5-diamino-2-[3-amino-6-(aminomethyl)-4.5-dihydroxyoxan-2-yl]oxy-6-hydroxycyclohexyl]oxyhexyl]-4-oxopentanamide	C₂₃H₄₅N₅O₈	7.81	520.34	A complex amino-sugar conjugate that may contribute to cell-wall reinforcement and immune signaling, as glycosylated amine derivatives are known to modulate defense via plant glycan recognition	Zhang et al. [23]
11	13.28	4-hydroxy-*N*-[5-[hydroxy-[1-hydroxy-4-[5-[hydroxy(1-hydroxyethyl)amino]pentylamino]-4-oxobutyl]amino]pentyl]-4-[hydroxy(methyl)amino]butanamide	C₂₁H₄₅N₅O₈	13.24	496.34	A polar amino-sugar-polyol derivative that may function as an osmoprotectant and redox modulator, and potentially act in sugar-amine immune signaling	Zhang et al. [23]; Trouvelot et al. [36]
12	16.38	(2S)-1-[(2S)-2-[[(2S.3S)-2-[[(2S)-2-[[(2S.3R)-2-[[(2 R.3S)-2-[[(2S)-2-[[2-[[(2S)-2-[acetyl(methyl)amino]pentanoyl]amino]acetyl]amino]-3-methylbutanoyl]amino]-3-methylpentanoyl]amino]-3-hydroxybutanoyl]amino]pentanoyl]amino]-3-methylpentanoyl]amino]-5-(diaminomethylideneamino)pentanoyl]-*N*-ethylpyrrolidine-2-carboxamide	C₄₉H₈₉N₁₃O₁₁	3.22	1.036.69	N/A	N/A
13	18.26	(2S)-2.6-diamino-*N*-[(2S)-6-amino-1-[[(2S)-6-amino-1-[[(2S)-1-[[(2S)-6-amino-1-[[(2S)-3-methyl-1-oxobutan-2-yl]amino]-1-oxohexan-2-yl]amino]-5-(diaminomethylideneamino)-1-oxopentan-2-yl]amino]-1-oxohexan-2-yl]amino]-1-oxohexan-2-yl]hexanamide	C₃₅H₇₁N₁₃O₆	4.52	770.57	Functions as a peptide-like compound that may contribute to antifungal activity through disruption of fungal cell membrane integrity	Volovik et al. [37]

## 4. Discussion

This study demonstrated that pre-exposure to UV-B irradiation effectively enhances resistance in shallot plants against *F. acutatum*, as evidenced by reduced disease incidence, severity, and disease progression (AUDPC). These findings support the notion that UV-B pre-exposure acts as a priming stimulus, whereby a controlled abiotic signal prepares plants to respond more efficiently to subsequent pathogen challenge. This priming effect is reflected not only in disease suppression but also in the maintenance of plant growth, physiological stability, and metabolic reprogramming under pathogen pressure.^38^

The reduction in disease parameters following UV-B treatment indicates that pre-exposure activates intrinsic antioxidant defense mechanisms and defense-associated physiological responses prior to pathogen challenge. UV-B radiation is known to trigger signaling pathways such as UVR8-mediated responses, which regulate defense-related gene expression and secondary metabolite biosynthesis.^39^ In this study, the lower AUDPC values in UV-B-treated plants suggest a delay and suppression of disease progression, highlighting enhanced resistance rather than mere tolerance. This finding aligns with previous reports that UV-B acts as an elicitor, promoting induced resistance through systemic acquired resistance-like mechanisms.^40^

Pathogen infection alone significantly impaired plant growth, as shown by reductions in plant height, biomass, and root development.^41^ These effects are typical of *Fusarium*-induced stress, which disrupts water and nutrient uptake and interferes with physiological processes. However, UV-B pre-exposure mitigated these negative effects, allowing plants to maintain relatively higher growth performance under infection.^42^ This suggests that UV-B-induced antioxidant and defense-associated responses do not impose a substantial growth penalty, which is often a limitation of stress-induced resistance strategies. Instead, UV-B appears to balance growth and stress adaptation, enabling plants to sustain biomass accumulation while resisting pathogen attack.

At the physiological level, UV-B pre-exposure enhanced photosynthetic pigment content, even under pathogen stress. The recovery of chlorophyll and carotenoid levels in UV-B + pathogen-treated plants indicates protection of the photosynthetic apparatus. Carotenoids, in particular, play a crucial role in photoprotection and ROS scavenging.^43^ ^,^ ^44^ The maintenance of pigment levels suggests that UV-B treatment preserves photosynthetic efficiency, thereby supporting energy availability for antioxidant and defense-associated physiological responses.

The accumulation of phenolic compounds further supports the activation of plant antioxidant and defense-associated biochemical responses. Phenolic compounds are widely recognized as important components of plant defense responses, contributing to antimicrobial activity, cell-wall reinforcement, antioxidant protection, and stress signaling. The highest phenolic content observed in the combined UV-B and pathogen treatment indicates a synergistic effect, where UV-B priming enhances the plant's capacity to mount a stronger antioxidant and defense-associated response upon infection.^45^ ^,^ ^46^ This primed response is a defining characteristic of induced resistance, characterized by faster and more robust activation of antioxidant and defense-associated pathways.

Similarly, the increased activities of antioxidant enzymes (CAT and POD) highlight the role of ROS regulation in UV-B-mediated antioxidant defense. While ROS function as signaling molecules in plant immunity, their excessive accumulation can cause oxidative damage. The elevated enzyme activities observed in UV-B-treated plants suggest an improved capacity to maintain redox homeostasis, preventing cellular damage while sustaining stress- and defense-associated signaling.^47^ The highest enzyme activities in the combined treatment further indicate that UV-B priming enhances the efficiency of ROS detoxification during pathogen attack.^48^

Metabolomic analysis provides additional insight into the biochemical responses associated with UV-B pre-exposure. The identification of metabolites related to stress signaling, redox regulation, and antimicrobial activity suggests that UV-B treatment induced extensive metabolic adjustments. Notably, the occurrence of polyamine derivatives, amino-sugar conjugates, and phenolic-related metabolites in UV-B-treated plants is consistent with the enhanced CAT and POD activities and increased phenolic accumulation observed in this study. These metabolite classes have previously been associated with ROS homeostasis, cell-wall remodeling, and stress adaptation.^49^ ^,^ ^50^

Beyond these general functions, the detected metabolite classes appear to contribute to distinct components of biotic defense. Polyamine derivatives are known to regulate ROS homeostasis and defense signaling, thereby facilitating antioxidant responses during pathogen infection. Amino-sugar conjugates are associated with cell-wall remodeling and immune signaling, which may strengthen structural barriers against pathogen invasion. Phenolic-related metabolites contribute to antioxidant protection, antimicrobial activity, and reinforcement of the cell wall. Conversely, quinazoline, benzimidazole, and hydrazine derivatives identified predominantly in pathogen-treated plants have been reported to possess antimicrobial or antifungal properties, suggesting the activation of defense-associated secondary metabolism in response to *F. acutatum*. Peptide-like metabolites detected in the combined UV-B+ pathogen treatment group may further contribute to defense by functioning as antimicrobial peptides or signaling molecules involved in plant immunity.^49^

Fold-change analysis (Supplementary Table S1) further demonstrated that UV-B selectively altered metabolite abundance rather than uniformly increasing all detected compounds. For example, the carbonic acid derivative detected at RT 1.28 increased approximately fourfold following UV-B treatment (FC = 4.06) and remained elevated under the combined UV-B+*F. acutatum* treatment (FC = 3.61), while the polyamine derivative detected at RT 5.01 also increased following UV-B treatment (FC = 1.23) and the combined treatment (FC = 1.47). In contrast, the amino-sugar conjugate detected at RT 12.66 decreased after UV-B exposure (FC = 0.73), indicating that UV-B redirected metabolic flux toward specific pathways rather than promoting general metabolite accumulation. Moreover, the quinazoline derivative detected at RT 11.60 showed a 1.32-fold higher abundance in the combined UV-B+*F. acutatum* treatment than in plants inoculated with *F. acutatum* alone, suggesting that UV-B priming further enhances pathogen-responsive secondary metabolism. Collectively, these findings indicate that UV-B pre-exposure selectively reprograms defense-related metabolism, reinforcing antioxidant regulation, antimicrobial capacity, and stress adaptation, which together contribute to the observed reduction in disease development.

**Figure 7.**
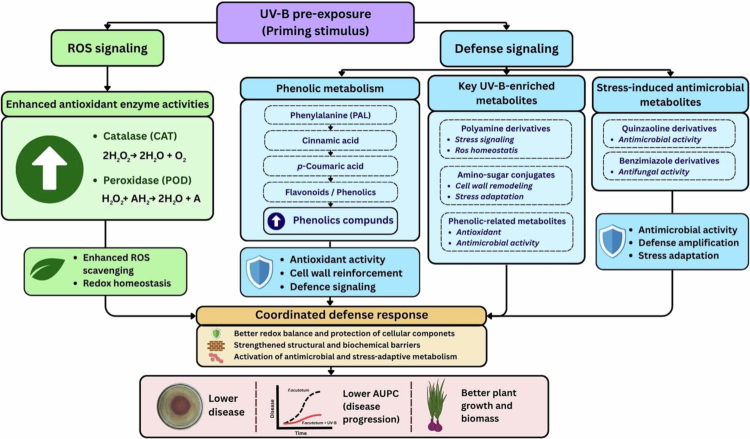
Proposed mechanism underlying UV-B-induced resistance against *F. acutatum* in shallot plants.

Taken together, the physiological, biochemical, and metabolomic responses observed in this study indicate that UV-B pre-exposure enhances shallot resistance against *F. acutatum* through coordinated antioxidant regulation, physiological protection, and metabolic reprogramming. A schematic model summarizing the proposed mechanism is presented in Figure 7, illustrating how UV-B pre-exposure activates antioxidant defenses, promotes phenolic metabolism, and modulates defense-associated metabolites, ultimately improving redox homeostasis, suppressing disease progression, and maintaining plant growth under pathogen stress. These findings highlight the potential of UV-B priming as a sustainable, non-chemical strategy for disease management in horticultural production while reducing reliance on chemical fungicides. Nevertheless, because metabolite identification was based on LC–MS/MS annotation and because the physiological parameters evaluated primarily reflect antioxidant-associated responses rather than the complete molecular defense network, the biological functions of individual metabolites remain putative. Future studies should incorporate additional defense-related enzymes, such as phenylalanine ammonia-lyase (PAL) and polyphenol oxidase (PPO), together with analyses of immune signaling pathways and defense-related gene expression to further validate the proposed mechanism of UV-B-induced resistance.

## 5. Conclusion

This study demonstrated that UV-B pre-exposure effectively reduced the development of twisted disease caused by *F. acutatum* in shallot plants. UV-B-treated plants exhibited lower disease incidence, disease severity, and AUDPC, while maintaining plant growth, photosynthetic pigment content, phenolic accumulation, and antioxidant enzyme activities (CAT and POD) under pathogen stress. LC–MS/MS analysis further revealed treatment-dependent changes in metabolite composition, with metabolites associated with stress signaling, redox regulation, and antimicrobial activity that were consistent with the observed physiological and biochemical responses.

Collectively, these findings indicate that UV-B pre-exposure enhances resistance to *F. acutatum* through coordinated antioxidant and metabolic responses that contribute to improved redox homeostasis and the suppression of disease development. Although the biological functions of individual metabolites require further experimental validation, this study provides new insight into the physiological and metabolomic responses associated with UV-B-induced resistance in shallot. These findings highlight the potential of UV-B pre-exposure as an environmentally friendly strategy for sustainable disease management and provide a foundation for future studies investigating the molecular mechanisms underlying UV-B-mediated resistance.

## Supplementary Material

Supplementary Material Supplementary Table S1.docx

## Data Availability

The data that support the findings of this study are available from the corresponding author, F.D., upon reasonable request.
