# Supplementary material for: Pre-exposure to UV-B irradiation induces antioxidant enzymes and mitigates Fusarium acutatum stress in shallot (Allium cepa L.)
Source: Plant Signal Behav. 2026 Aug 15;21(1):2716981. doi: 10.1080/15592324.2026.2716981 (PMC13479827; doi:10.1080/15592324.2026.2716981)
Supplement: Supplementary file 1 [file KPSB_A_2716981_SM2985.docx]

**Supplementary Table S1.** Relative peak area (%) and fold-change (FC) of putatively identified non-volatile metabolites detected by LC–MS/MS in shallot leaves under different treatments.

| **No.** | **RT (min)** | **Putative metabolites** | **Molecular formulas** | **Control areas (%)** | ***F. acutatum* areas (%)** | **UV-B areas (%)** | **UV-B + *F. acutatum* areas (%)** | **FC (UV-B / Control)** | **FC (F / Control)** | **FC (UV-B + F / F)** | **FC (UV-B + F / Control)** |
| --- | --- | --- | --- | --- | --- | --- | --- | --- | --- | --- | --- |
| 1 | 1.28 | Carbonic acid; 2-ethyl-2-(hydroxymethyl)propane-1,3-diol | C₉H₂₀O₁₂ | 1.59 | 6.51 | 6.46 | 5.74 | 4.06 | 4.09 | 0.88 | 3.61 |
| 2 | 2.09 | 3-[2-[2-[(2-hydrazinyl-2-oxoethyl)carbamoyl]hydrazinyl]-2-oxoethyl]-1,1-dimethylurea | C₈H₁₇N₇O₄ | 15.67 | ND | 17.28 | ND | 1.10 | ND | ND | ND |
| 3 | 2.97 | [(2R,3S,4R,5R)-5-(6-aminopurin-9-yl)-3,4-dihydroxyoxolan-2-yl]methylurea | C₁₁H₁₅N₇O₄ | 4.63 | ND | 5.69 | 4.65 | 1.23 | ND | ND | 1.00 |
| 4 | 4.60 | 6,10-dimethyldodecan-6-ol | C₁₄H₂₉O | 1.40 | 1.32 | 1.30 | 1.64 | 0.93 | 0.94 | 1.24 | 1.17 |
| 5 | 5.01 | 1-[N'-[3-[4-[3-[[amino-(prop-2-enylcarbamoylamino)methylidene]amino]propylamino]butylamino]propyl]carbamimidoyl]-3-prop-2-enylurea | C₂₀H₄₀N₁₀O₂ | 1.16 | ND | 1.43 | 1.70 | 1.23 | ND | ND | 1.47 |
| 6 | 5.39 | Hexadecyl(dipropyl)oxidanium | C₂₂H₄₇O | 0.86 | ND | 0.76 | 1.16 | 0.88 | ND | ND | 1.35 |
| 7 | 8.29 | 4-(4-pentylcyclohexyl)benzoic acid | C₁₈H₂₆O₂ | 1.16 | 1.32 | ND | ND | ND | 1.14 | ND | ND |
| 8 | 11.60 | 7-ethyl-6-methoxy-2-(4-methyl-1,4-diazepan-1-yl)-N-(1-methylpiperidin-4-yl)quinazolin-4-amine | C₂₃H₃₆N₆O | 1.79 | 2.22 | ND | 2.92 | ND | 1.24 | 1.32 | 1.63 |
| 9 | 11.87 | (2S)-N-[5-[[(2S)-2-amino-6-[3-[[(2S)-2-amino-5-(diaminomethylideneamino)pentanoyl]amino]propylamino]hexanoyl]amino]pentyl]-2-[[2-(1H-indol-3-yl)acetyl]amino]butanediamide | C₃₄H₅₈N₁₂O₅ | 3.61 | ND | ND | 5.81 | ND | ND | ND | 1.61 |
| 10 | 12.66 | *N*-[6-[3,5-diamino-2-[3-amino-6-(aminomethyl)-4,5-dihydroxyoxan-2-yl]oxy-6-hydroxycyclohexyl]oxyhexyl]-4-oxopentanamide | C₂₃H₄₅N₅O₈ | 6.13 | ND | 4.46 | ND | 0.73 | ND | ND | ND |
| 11 | 13.28 | 4-Hydroxy-*N*-[5-[hydroxy-[1-hydroxy-4-[5-[hydroxy(1-hydroxyethyl)amino]pentylamino]-4-oxobutyl]amino]pentyl]-4-[hydroxy(methyl)amino]butanamide | C₂₁H₄₅N₅O₈ | 10.66 | ND | 9.68 | 13.24 | 0.91 | ND | ND | 1.24 |
| 12 | 16.38 | (2R)-1-[[(1S,2S)-2-[[(2R)-2-hydroxy-9-phenylnonyl]-methylamino]cyclohexyl]-methylamino]-9-phenylnonan-2-ol | C₃₈H₆₂N₂O₂S | 4.47 | 3.05 | 3.48 | 3.22 | 0.78 | 0.68 | 1.06 | 0.72 |
| 13 | 17.49 | 18-[2-[2-[2-[2-[2-[2-[[(5S)-5-carboxy-5-[[3-[3-(1H-imidazol-5-yl)propanoylamino]-3-methyl-2-oxobutyl]amino]pentyl]amino]-2-oxoethoxy]ethoxy]ethylamino]-2-oxoethoxy]ethoxy]ethylamino]-18-oxooctadecanoic acid | C₄₇H₈₃N₇O₁₃ | 1.70 | 5.14 | ND | ND | ND | 3.02 | ND | ND |
| 14 | 17.91 | tert-butyl 4-[11-[[(2S)-5-[(2-methylpropan-2-yl)oxy]-2-[4-[5-[(2-methylpropan-2-yl)oxycarbonylamino]pentyl]triazol-1-yl]-5-oxopentanoyl]amino]undecanoyl]piperazine-1-carboxylate | C₄₁H₇₃N₇O₈ | 1.43 | ND | ND | ND | ND | ND | ND | ND |
| 15 | 18.44 | ethyl 4-[[6-[bis(3-methylbutyl)carbamoyl]-1-[3-[methyl(pentyl)amino]propyl]benzimidazol-2-yl]amino]piperidine-1-carboxylate | C₃₅H₆₀N₆O₃ | 17.92 | 11.89 | 6.62 | ND | 0.37 | 0.66 | ND | ND |

**Note:** ND, not detected.
